# Landscape of prognosis and immunotherapy responsiveness under tumor glycosylation-related lncRNA patterns in breast cancer

**DOI:** 10.3389/fimmu.2022.989928

**Published:** 2022-09-15

**Authors:** Wenchang Lv, Yufang Tan, Xiaomei Zhou, Qi Zhang, Jun Zhang, Yiping Wu

**Affiliations:** ^1^ Department of Plastic and Cosmetic Surgery, Tongji Hospital, Tongji Medical College, Huazhong University of Science and Technology, Wuhan, China; ^2^ Department of Thyroid and Breast Surgery, Shenzhen Qianhai Shekou Free Trade Zone Hospital, Shenzhen, China

**Keywords:** breast cancer, glycosyltransferase, lncRNA, risk score, prognosis, immunotherapy sensitivity

## Abstract

Aberrant glycosylation, a post-translational modification of proteins, is regarded to engage in tumorigenesis and malignant progression of breast cancer (BC). The altered expression of glycosyltransferases causes abnormal glycan biosynthesis changes, which can serve as diagnostic hallmarks in BC. This study attempts to establish a predictive signature based on glycosyltransferase-related lncRNAs (GT-lncRNAs) in BC prognosis and response to immune checkpoint inhibitors (ICIs) treatment. We firstly screened out characterized glycosyltransferase-related genes (GTGs) through NMF and WGCNA analysis and identified GT-lncRNAs through co-expression analysis. By using the coefficients of 8 GT-lncRNAs, a risk score was calculated and its median value divided BC patients into high- and low-risk groups. The analyses unraveled that patients in the high-risk group had shorter survival and the risk score was an independent predictor of BC prognosis. Besides, the predictive efficacy of our risk score was higher than other published models. Moreover, ESTIMATE analysis, immunophenoscore (IPS), and SubMAP analysis showed that the risk score could stratify patients with distinct immune infiltration, and patients in the high-risk group might benefit more from ICIs treatment. Finally, the vitro assay showed that MIR4435-2HG might promote the proliferation and migration of BC cells, facilitate the polarization of M1 into M2 macrophages, enhance the migration of macrophages and increase the PD-1/PD-L1/CTLA4 expression. Collectively, our well-constructed prognostic signature with GT-lncRNAs had the ability to identify two subtypes with different survival state and responses to immune therapy, which will provide reliable tools for predicting BC outcomes and making rational follow-up strategies.

## Introduction

In 2020, 2.3 million new breast cancer (BC) cases and 680 thousand new deaths are identified in women, making BC surpass lung cancer as the most commonly diagnosed female cancer and the leading cause of cancer death (1). In recent years, immunotherapy, especially the application of immune checkpoint inhibitors (ICIs) has revolutionized the treatment of BC patients (2). In many clinical trials, targeting programmed cell death-1 (PD-1)/programmed cell death ligand-1 (PD-L1) and cytotoxic T-lymphocyte antigen-4 (CTLA-4), has yielded favorable clinical efficacy in BC patients (3). Nevertheless, some patients may show less benefit from ICIs, especially those with ER-positive subtype (4). Hence, it is crucial to search for an effective method to predict BC patients’ long-term survival and response to ICI treatment.

Glycosylation is a multistep process of post-translational modification of proteins that removes and adds individual carbohydrates to proteins and lipids by exploiting 200 glycosyltransferase enzymes (5). Protein glycosylation includes N-linked glycosylation, O-linked glycosylation, C-mannosylation, phospho-glycosylation and glypiation (6). Abnormal and disordered glycosylation is associated with cancer and various disease, as it engages in uncontrolled cell proliferation, migration and differentiation (7). Many studies have unraveled that tumor cells show extensive protein glycosylation in comparison with their non-malignant counterparts (8, 9). Cancer-associated glycosylation changes, mainly including O-glycan truncation, sialylation, fucosylation, and N-glycan branching, are directly involved in angiogenesis, immune modulation, epithelial-mesenchymal transition (EMT) and metastasis (10). Fang et al. uncovered that the aberrant initiation of O-glycosylation regulated by LAMTOR5 conduced to BC distant metastasis (11). Notably, N-glycosylation of PD-L1 could keep its stability and progressively interact with PD-L1, thus allowing BC cells to avoid immune surveillance (12, 13). Thus, altered glycosylation is not only a contributor to malignant transformation in cancer but also a regulator of ICIs in the immune response.

Several prognostic models based on glycosylation-related genes have been emerging for predicting cancer prognosis. For example, Zhao et al. harnessed 4 glycosylation-related mRNAs to calculate the risk score in ovarian cancer, and the risk score was negatively correlated with tumor purity and tumor mutation burden, which suggested that low-risk scores might predict a better benefit from ICI treatment (14). Our previous study screened 9 glycosyltransferase genes to develop a prognostic signature in BC, and the high-risk-group patients showed shorter survival probability and more immunosuppressive profile (15). However, there are no reports on establishing prognostic signature based on glycosylation-related long non-coding RNAs (lncRNAs) for predicting BC prognosis and response to ICI therapy. Some distinct characteristics of lncRNAs from mRNAs, such as higher tissue specificity, developmental stage specificity and cell subtype specificity (16), determine the different effects of lncRNAs in cancer development and tumor microenvironment (TME). LncRNAs have been reported to engage in various regulatory processes, including cell growth, proliferation, differentiation, apoptosis, motility and invasion, signal transduction, DNA damage regulation, immune response, and pluripotency (16). Therefore, it is promising to identify a signature of glycosylation-related lncRNAs for a more accurate prediction of BC long-term survival and response to ICI treatment.

In the present, we firstly screened out glycosyltransferase-related genes (GTGs) mostly associated with BC prognosis through the nonnegative matrix factorization (NMF) method and weighed gene co-expression network analysis (WGCNA). Glycosyltransferase-related lncRNAs (GT-lncRNAs) were stepwise identified *via* co-expression analysis. Then, targeted GT-lncRNAs were selected to calculate the risk score according to univariate, LASSO and multivariate cox regression analyses. The median risk score divided BC samples into high-risk and low-risk groups, which displayed differential overall survival (OS), progression-free survival (PFS), disease-free interval (DFI), clinical features, immune infiltration, response to ICI treatment and chemo-drugs sensitivity. In summary, our study will provide novel insights into the precision treatment for BC on the basis of glycosylation.

## Materials and methods

### Data collection

A total of 1089 BC cases with corresponding mRNA expression data (FPKM format), lncRNA expression data and clinical data, were downloaded from the TCGA data portal (https://portal.gdc.cancer.gov/).

### Molecular subtypes based on GTGs

Firstly, a total of 169 GTGs were extracted from the TCGA expression profile data. Then, 1089 BC samples were clustered by performing the NMF analysis according to the “brunet” standard and 50 iterations. The k value, the number of clusters, was set as 2-10. The average contour width of the common member matrix was determined by using the NMF package in R, and the minimum member numbers of each subtype were set to 10. Finally, the optimal number of clusters was identified according to indexes, including cophenetic, dispersion, and silhouette.

### WGCNA analysis

The WGCNA algorithm was applied to excavate the co-expressing coding genes and co-expression modules by establishing the scale-free co-expression network (17). Firstly, based on the expression profiles of 169 GTGs, the Pearson correlation coefficient between two genes and adjacency function was calculated by the R package WGCNA. The parameter β, the soft threshold of the adjacency matrix, was used to construct scale-free networks to emphasize the strong correlations between GTGs. Then, the adjacency matrix was transformed into a topology matrix, and the topological overlap measure (TOM) was used to describe the degree of association between genes. Next, the hierarchical clustering analysis was used to calculate modules with the distance as 1-TOM. The minimum size of the module was set as 30. Finally, the module eigengenes were linked to subtypes in the present study, including clinical characteristics (survival state, age, stage and TNM stage), and cluster subtypes based on GTGs (C1 and C2). The module with the highest correlation and gene significance with C1 and C2 was selected for the next analysis, and these genes were identified as hub GTGs. Moreover, the clusterProfiler R package 3.42.0 was used to perform Gene ontology (GO) and Kyoto Encyclopedia of Genes and Genomes (KEGG) functional enrichment analyses of these hub GTGs to annotate the molecular functions of the selected module genes.

### Construction and validation of the risk score based on GT-lncRNAs

After the acquisition of 169 hub GTGs, GT-lncRNAs were identified through co-expression analysis with GTGs from the TCGA project. The GT-lncRNAs associated with BC prognosis were selected *via* the univariate and multivariate Cox regression analysis with R package “survival”. LASSO Cox regression was analyzed with “glmnet” package to reduce the number of genes and obtain the most predictive genes. The risk score was calculated depending on the coefficients of GT-lncRNAs and according to the following formula:


$$Risk\;score=\sum_{i=1}^{n}\left(\beta i*Expi\right)$$

Where Expi is the expression of GT-lncRNAs and βi is the coefficient.

The Kaplan-Meier method was used to compare the differences in OS, DFS and PFI between different groups. The accuracy of the risk model was evaluated by using Receiver operating characteristic (ROC) analysis with R package “survivalROC”. The nomogram was established to predict the 1-, 2-, and 3-year survival rates of BC patients by using the “rms” R package. The predictive performance of the risk score was compared with other signatures *via* the concordance index (C-index) in the “rms” R package. A Sankey diagram was generated to visualize the assignment of different variables by using the R package “networkD3”.

### Gene set enrichment analysis (GSEA) and immunity analysis

GSEA software (http://software.Broadstitute.org/GSEA/) was used to explore the potential differences in the biological function and signaling pathways between the two risk groups based on the KEGG and GO gene sets in the TCGA project. The threshold for significantly enriched functional annotations was set as p< 0.05 and false discovery rate (FDR)< 0.25. The infiltrating scores of 22 immune cells and the activities of 13 immune-related pathways were quantified by using single-sample gene set enrichment analysis (ssGSEA) with the “gsva” R package. The abundance of immune cells and stromal cells for each BC sample was evaluated by using the immune score, tumor purity, ESTIMATE score, and stromal score with the ESTIMATE algorithm. The box plots were drawn to visualize the differences.

### The cancer-immunity cycle analysis

The cancer-immunity cycle, a cyclical process in the immune system to eradicate cancer, is a vital framework for tumor immunotherapy study (18). The cycle mainly includes seven steps (19) (1): release of cancer antigen (2), cancer antigen presentation (3), initiation and activation (4), trafficking T cells to the tumor (5), infiltration of T cells into the tumor (6), T cell recognition of cancer cells, and (7) T cell killing of cancer cells. The information of genes from each step was downloaded from Tracking Tumor Immunephenotype (http://biocc.hrbmu.edu.cn/TIP/index.jsp). Furthermore, the ssGSEA algorithm was used to quantify the scores of the seven steps. The differences in the scores of the seven steps in immunophenotyping were compared between high- and low-risk groups.

### The immunotherapy response and chemo-drug sensitivity analysis

The TIDE algorithm was used to evaluate the response to ICI therapy. Immunophenoscore (IPS), constituted of four parts (effector cells, immunosuppressive cells, MHC molecules, and immunomodulators), determines the immunogenicity and reflects the response of the patients to immunotherapy (20). The IPS of all BC patients were obtained from The Cancer Immunome Atlas (TCIA) (https://tcia.at/home). Using the gene expression of various immune molecules could calculate the IPS, which ranged from 0 to 10. BC patients’ responses to anti-PD1 and anti-CTLA-4 treatment. The responses to anti-PD-1 and anti-CTLA-4 treatment in BC patients with high and low risk were assessed by utilizing the SubMAP algorithm. The data of half-inhibitory concentration (IC50) of commonly used chemotherapeutic drugs in BC patients were obtained from the TCGA project by using the “pRRophetic” and “ggplot2” R package.

### Cell culture and si-MIR4435-2HG transfection

MCF-7 and MDA-MB-231 were obtained from American Type Culture Collection (Manassas, VA, USA), and cultured at 37°C in a 5% CO2 atmosphere with Dulbecco’smodified Eagle’smedium (DMEM; Gibco, Carlsbad, CA, USA) supplemented with 10% fetal bovine serum (FBS; Gibco, Carlsbad, CA, USA). The small interference RNAs (siRNAs) targeting MIR4435-2HG and negative control were designed and synthesized by Ribo Biotech (Guangzhou, China). By using Lipofectamine 3000 Transfection Reagent (Invitrogen, CA, USA), siRNAs were transfected into MDA-MB-231 and MCF-7 cells. Then, using RT-PCR to estimate transfection efficiency after 24 h. Total RNAs were extracted from cultured BC cells by TRIzol (Takara, Japan), and cDNA was synthesized by the Strand cDNA Synthesis Kit (Yeasen, Shanghai, China) according to the manufacturer’s protocols. The qRT-PCR was performed with QuantStudio1 (ABI Q1, USA) by using the SYBR GreenTM Master Mix (Yeasen, Shanghai, China). All primer sequences used for qRT-PCR were provided in **Table S1**.

### Co-culture of BC cells and M0 macrophages by transwell assay

For the si-MIR4435-2HG mediated the effect of BC cells on macrophage polarization. The BC cell lines (MDA-MB-231 and MCF-7), transfected with si-control or si-MIR4435-2HG, were plated into the upper chamber. THP-1 cells were plated into the lower chamber. The upper and lower chambers were filled with the same DMEM medium. After 48 h co-culture, qRT-PCR was used to detect the relative mRNA levels of ARG1 (M2 macrophage marker) and iNOS (M1 macrophage marker), in the cells of the lower chamber.

For the effects on macrophage migration. THP-1 cells were plated into the upper chamber. The BC cell lines (MDA-MB-231 and MCF-7), transfected with si-control or si-MIR4435-2HG, were plated into the lower chamber. The upper and lower chambers were filled with the same DMEM medium. After 48 h co-culture, the upper chamber was washed with phosphate-buffered saline (PBS) twice, fixed with 4% paraformaldehyde for 30 min, stained with 0.1% crystal violet for 10 min and counted by a microscope and ImageJ software.

### Cell proliferation and migration assays

Cell Counting Kit-8 (CCK-8) assay (Yeasen, Shanghai, China) was used to estimate cell proliferation. Cells were seeded in a 96-well plate at 2 × 103/well density. Until the confluence reached 40%, the culture cells were transfected with si-MIR4435-2HG and then, 10 μL CCK-8 reagent was directly added to each well at the specified time (0, 24 h) and then incubated at 37°C for 1.5 h. The optional density (OD) was obtained at 450 nm by a microplate reader (BioTek Instruments, VT, USA).

Scratch/Wound healing assay was carried out to explore the migration abilities of BC cells. MCF-7 and MDA-MB-231 cells were plated on the 6-well plate after transfection. Until the cells were grown to 90% confluence to form a cell monolayer, a 200 μL micropipette tip was used to create a straight scratch on the single-cell layer in each well. After washing away detached cells and debris with PBS, the cells were cultured at 37°C for 24 h in a serum-free DMEM medium. The horizontal distances of the migrating cells were captured by microscopy and were calculated by ImageJ software.

The migration performance of BC cells was also identified *via* transwell assay by using a 24-well culture plate (8-mm pore size; Corning, USA). MDA-MB-231 cells and MCF-7 cells were plated into the upper chambers in 200 μL serum-free DMEM, and 500 μL DMEM medium supplemented with 20% FBS was placed into the bottom chambers as an attractant. After a culture at 37°C for 24 h, the migrated cells on the chamber membranes were fixed with 4% paraformaldehyde for 30 min, stained with 0.1% crystal violet for 10 min, and counted by a microscope and ImageJ software.

### Statistics analysis

All statistical analysis was performed by using R version 4.0.5 and GraphPad Prism (version 8.0). The independent t test was utilized to compare the continuous variables between the two groups, and the χ2-test was used to compare the differences in proportions. The univariate and multivariate Cox proportional hazards regression analyses were used to evaluate the prognostic ability. The Kaplan-Meier analysis through the log-rank test was used to compare the OS, DFS and PFI between different groups. The Wilcoxon test was performed to compare IC50 values between groups. The correlation between two variables was evaluated by Pearson correlation analyses. All statistical tests were two-sided, and the values of P< 0.05 were considered to be significant.

## Results

### Identification of molecular subtypes by NMF analysis

**Figure 1:**
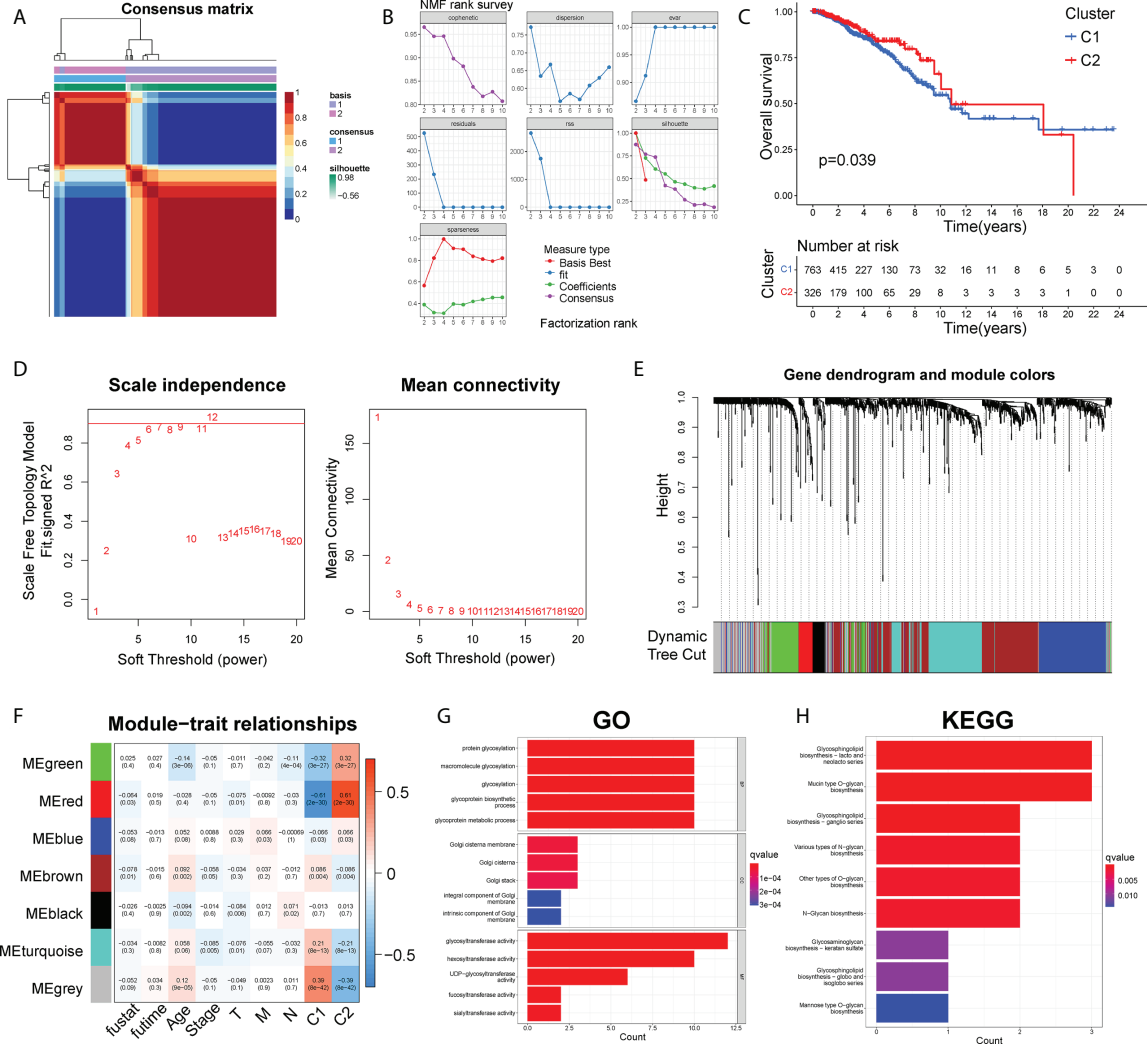
The NMF and WGCNA analysis to identify functional module. **(A)** Consensus map of NMF clustering. **(B)** The cophenetic, rss, and dispersion distributions. **(C)** The Kaplan-Meier curves about OS of C1 and C2 clusters. **(D)**. Cluster analysis of the samples. **(E)** Gene dendrogram and module colors. **(F)** Correlations of modules with clinical phenotypes. **(G)** Top 10 results for GO enrichment of genes in the grey module. **(H)** Top 10 results of KEGG enrichment of genes in the grey module.

The NMF was firstly used to cluster all 1089 BC patients and 169 glycosyltransferase-related gene expression profiles (**Table S2**) from the TCGA project based on 50 iterations and brunet criterion (21). K, the number of clusters, was set as 2-10. The R package NMF was used to identify the average contour width of the common member matrix. The minimum number of members of each subtype was 10. Then, according to cophenetic, dispersion, silhouette and sparseness, the best value of k was determined as 2 (**Figures 1A, B**). Compared with the C2 subgroup, BC patients in cluster1 had a shorter survival time and poor prognosis (P< 0.05) (**Figure 1C**).

### Identification of functional module by WGCNA analysis

Firstly, by calculating the Pearson correlation coefficient between two genes, the expression data of genes was used to establish the similarity matrix. Then, the similarity matrix was converted into an adjacency matrix and stepwise transformed into a topology matrix, which described the degree of association between genes by using the TOM. The power of β = 6 was set as the soft-thresholding parameter to satisfy the scale-free topology (R^2^ > 0.9) (**Figure 1D**). 1-TOM was determined as the distance to cluster the genes, and then according to the cutoff value = 0.25, the hierarchical clustering tree was constructed (**Figure 1E**). The eigengene of each module was calculated and the closer modules were merged into new modules. Finally, a total of 7 gene modules of GTGs were identified, with the minimum size of the gene group = 15, height =0.5, and deepSplit =2 (**Figure 1F**). The gray module was identified as a group of genes that could not be clustered into other modules. Furthermore, we explored the correlations between each module and survival outcome, age, stage, TNM stages and cluster1, cluster2. The results found that the most significant module of cluster1 and cluster2 was both grey (**Figure 1F**). The grey module of cluster1 contained 100 genes, and grey module of cluster2 contained 108 genes. Therefore, the combined grey modules of cluster1 and cluster2 contained a total of 208 genes.

Besides, the KEGG pathway enrichment and GO analysis were performed to analyze the potential biological function and enriched pathways of the above 208 genes. Notably, those genes were mainly associated with biological processes about glycosylation and the molecular function of glycosyltransferase activity (**Figure 1G**). The top 9 KEGG pathways were shown in **Figure 1H**, showing that those 208 genes were mainly enriched in pathways about glycan biosynthesis.

### Construction of the prognostic risk model

Grouping of the training set and testing set. All 1089 BC samples from the TCGA project were identified as the entire set. 545 BC patients were randomly selected to serve as the internal testing set. 544 BC patients were identified as the training set. The inclusion of patients was according to the following conditions: 1) The patients were diagnosed as BC; 2) The case had integral expression profiles and clinical information. The exclusion of patients was according to the following conditions: 1) The patients without survival information or survival time was less than 30 days; 2) The patients without clinical staging or pathological grade information. **Table S3** showed the clinical characteristics of BC patients from the TCGA project in the training, testing and entire set.

Identification of GT-lncRNAs and establishment of the risk score. Through co-expression analysis, 200 GT-lncRNAs were obtained from the TCGA project. The univariate Cox regression analysis was firstly executed in the training set to select GT-lncRNAs significantly associated with BC prognosis (P< 0.05), and 13 lncRNAs were identified (**Table S4**). To further reduce the number of lncRNAs and make the prognostic model more accurate and predictive, the LASSO Cox regression analysis was performed. The model tended to be stable and optimal when lambda = 0.06 (**Figure S1A**). Hence, 12 lncRNAs were confirmed as targeted genes (**Table S5**). Stepwise, the multivariate Cox regression analysis was conducted on these 12 lncRNAs, and 8 lncRNAs were finally utilized to construct the risk model (**Figures S1A, B**; **Table S6**). The coefficients of these 8 lncRNAs were used to calculate the risk score of both the training and testing set. The formula was as followed:


$$Risk\;score\;=\;Exp_{MIR4435-2HG}*\;\left(0.121211\right)\;+\;Exp_{MAPT-AS1}*\;\left(-0.22895\right)\;+\;Exp_{TGFB2-AS1}*\;\left(-0.17321\right)\;+\;Exp_{AL357054.4}*\;\left(-0.28143\right)\;+\;Exp_{AL161719.1}*\;\left(0.129371\right)\;+\;Exp_{OTUD6B-AS1}*\;\left(0.085594\right)\;+\;Exp_{AC083799.1}*\;\left(-0.03219\right)\;+\;Exp_{LINC01016}*\;\left(-0.06306\right)$$

**Figure 2:**
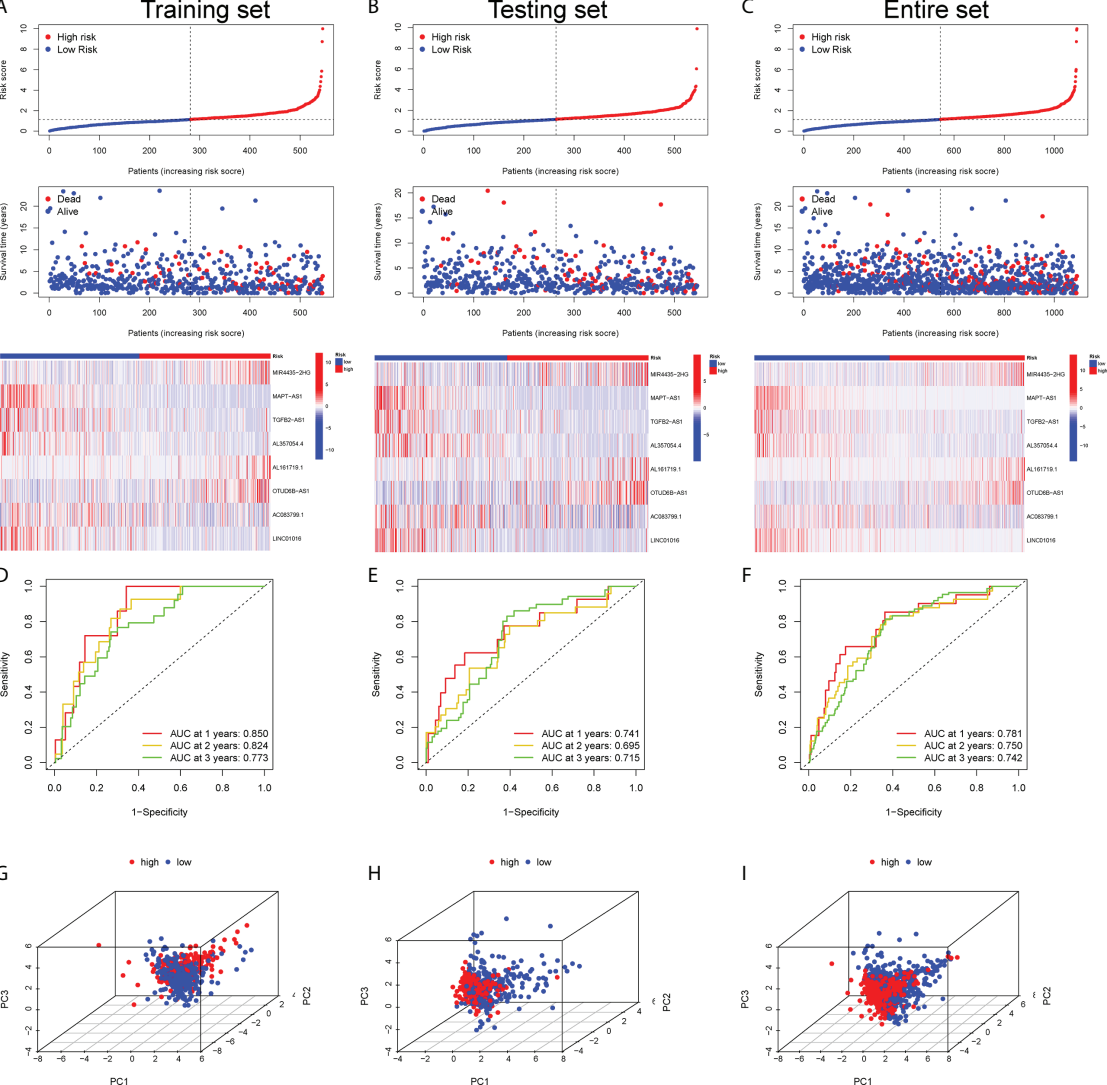
Prognostic validation of the risk score in the training, testing and entire set. The scatter plot of every sample and the heatmap of the expression profiles between two risk subgroups in the training set **(A)**, testing set **(B)** and entire set **(C)**. The AUC value of the risk score at 1-, 2- and 3-year survival in the training set **(D)**, testing set **(E)** and entire set **(F)**. The PCA visualizing the distribution pattern of the high- and low-risk patients in the training set **(G)**, testing set **(H)** and entire set **(I)**.

By calculating the risk score of each BC sample according to the expression of 8 GT-lncRNAs, BC patients in the training, testing and entire set could be divided into the high-risk and the low-risk group based on the median threshold of risk score. In addition, the risk score distribution of the sample showed that the proportion of deaths of samples with high-risk scores was significantly higher than that with low-risk scores, which proposed that the high-risk score predicted wore prognosis of BC patients. And the heatmap visualized the expression of 8 GT-lncRNAs between high- and low-risk groups (**Figures 2A–C**). Both in the training and testing set, the Kaplan-Meier curves unraveled that BC patients in the high-risk group had shorter OS, DFS and PFI than those in the low-risk group (**Figures S2A, B**). In the training set, the AUC value was 0.850, 0.824 and 0.773 at 1-, 2- and 3-year survival (**Figure 2D**). In the testing set, the AUC value was 0.741, 0.695 and 0.715 at 1-, 2- and 3-year survival (**Figure 2E**). In the entire set, the AUC value was 0.781, 0.750 and 0.742 at 1-, 2- and 3-year survival (**Figure 2F**). The PCA showed distinct distributions of two subgroups (**Figures 2G–I**).

### Validation of the prognostic value of the risk score

In the univariate Cox regression analysis, age, clinical stage, TNM stage and risk score were significantly associated with the survival of BC patients in the training and testing cohort (P< 0.001) (**Table S7**). Nevertheless, in the multivariate Cox regression analysis, only the age, clinical stage and risk score were significantly related to the BC prognosis in the training and testing cohort (P< 0.05) (**Table S8**). These results demonstrated that the risk score could act as an independent predictor in BC prognosis. Then, both in the training and testing cohort all independent factors, including age, clinical stage, TNM stage and the risk score, were recruited to construct a nomogram for predicting the 1-, 3- and 5-year OS of individual BC patients (**Figures S3A, B**). The calibration plot for the prediction about 1-, 3- and 5-year OS showed ideally consistency with actual observation in the training set, indicating the optimal predictive effect of the nomogram and excellent prognostic value of the risk score (**Figures S3A, B**). The ROC curve exhibited that the AUC value of the risk score at 1-, 2-, and 3-year OS reached 0.781, 0.751 and 0.742, respectively (**Figure S3C**). Moreover, the AUC value of the risk score at 1-year OS was just below age, while higher than clinical stage and TNM stages (**Figure S3D**). The time-dependent C-index curve unraveled that the risk score performed better than age, clinical stage and TNM stages in predicting BC prognosis (**Figure S3E**).

### External comparison of the risk model with other models

To further verify the reliability and stability of the risk model, the other external 4 prognostic models were selected for comparison with our risk model, including Ping’s signature (22), Luo’s signature (23), Zhang’s signature (24) and Zhao’s signature (25). The method of calculating the risk score for all those external risk models was consistent with ours, and all those external risk models were applied for predicting BC prognosis. Intriguingly, the AUC value of our risk model at 5 years was higher than all other 4 external models (**Figures S4A–D**). The Kaplan-Meier analysis confirmed that except for Zhang’s signature, BC patients in the high-risk group displayed a shorter survival time in the other 3 models (**Figures S4E–H**). Notably, the C-index of our risk model was the highest (**Figure S4I**), indicating that the predictive effect of our risk model was more excellent than others. The corresponding HR and P value were displayed by the restricted mean survival (RMS) curve (**Figure S4J**).

### Predictive ability of the risk score in clinical outcomes and BC subtypes

**Figure 3:**
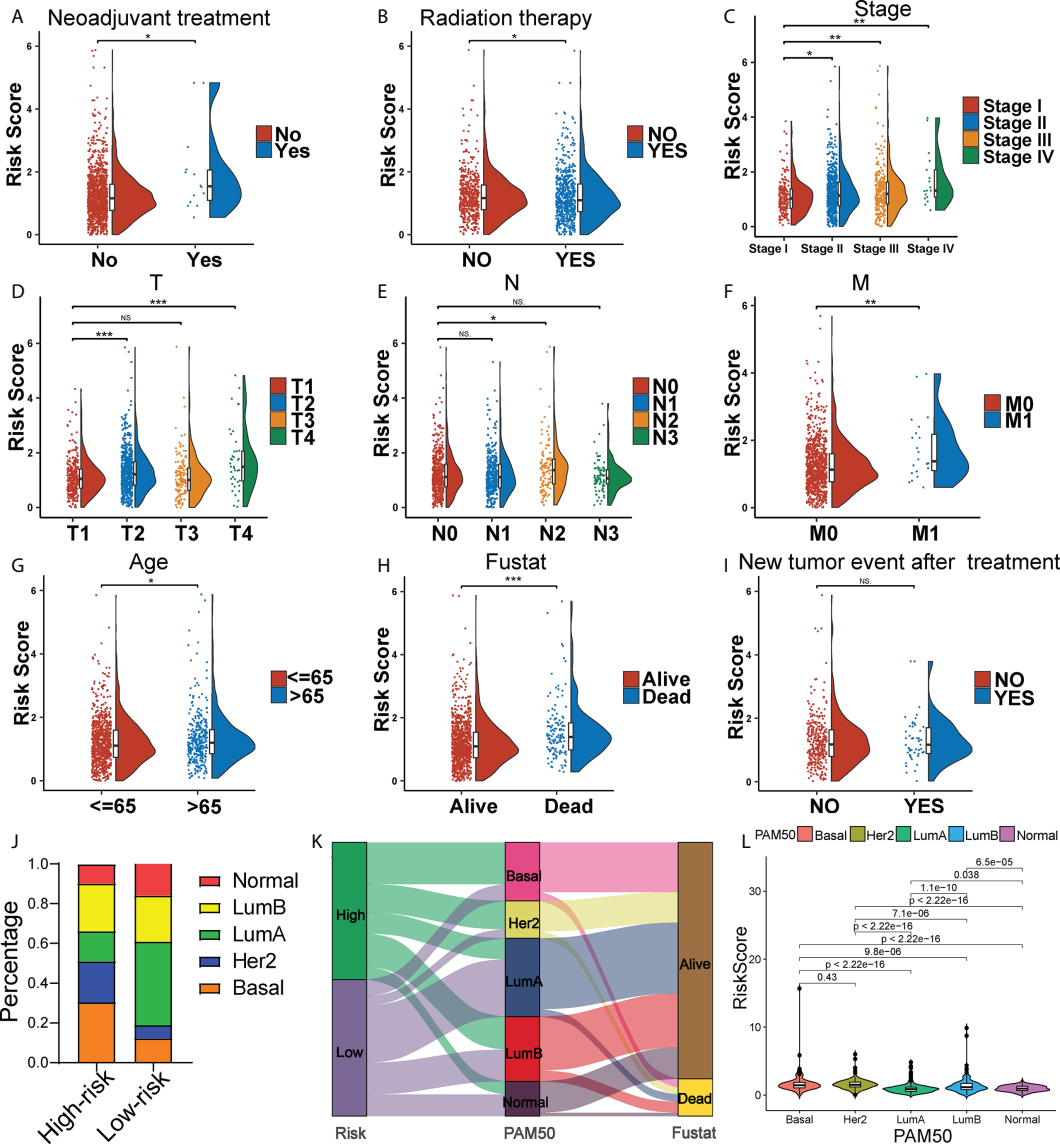
Clinical correlation of the risk score. The Raincloud plot showing the expression level of the risk score of the two risk group BC patients under neoadjuvant treatment **(A)**, and radiation therapy **(B)**, and in clinical stages **(C)**, T stages **(D)**, N stages **(E)**, M stages **(F)**, age groups **(G)**, death groups **(H)**, and new tumor event after treatment **(I)**. **(J)** The landscape of high- and low-risk-group patients at different BC subtypes. **(K)** Sankey diagram of distribution in subgroups with different risk scores, BC subtypes and survival states. **(L)** Box plots representing risk scores among all BC molecular subtypes. *P < 0.05, **P < 0.01; ***P < 0.001; ns, no significance.

It found that the risk score was higher in BC patients receiving neoadjuvant treatment while not under radiation therapy (**Figures 3A, B**). Moreover, BC patients with more advanced clinical and TNM stages were typically endowed with higher risk scores (**Figures 3C–F**). Meanwhile, patients with age > 65 and death, apt to adverse survival outcomes, also got higher risk scores (**Figures 3G, H**). However, the occurrence of new tumor events after treatment showed no significance in risk scoring (**Figure 3I**). Apparently, the risk score was an underlying predictor in BC patient clinical outcomes. Unraveling the potential correlation of the risk score with patients under different clinical characteristics and treatments might be an effective supplementary in predicting BC’s long-term prognosis. In addition, given the high heterogeneity of BC, we further explored whether BC patients with different molecular subtypes got different risk scores. Except for BC patients in the Her2 subtype, BC patients in basal-like type, luminal-A type, luminal-B type and normal-like type with higher risk scores generally had shorter OS in the Kaplan-Meier analysis (**Figure S5**). Intriguingly, the panorama of BC subtypes was dramatically different between high- and low-risk groups (**Figure 3J**). Particularly, high-risk patients were mainly distributed to the basal-like subtype (30%), while low-risk patients were mainly distributed to the luminal-A subtype (48%). The Sankey diagram showed the assignment based on the risk score, PAM50 classification and survival status of BC patients (**Figure 3K**). BC patients with different PAM50 subtypes significantly got distinct risk scores (**Figure 3L**), demonstrating the possible application of the risk score in distinguishing BC subtypes.

### Immunological landscape in two risk subtypes

**Figure 4:**
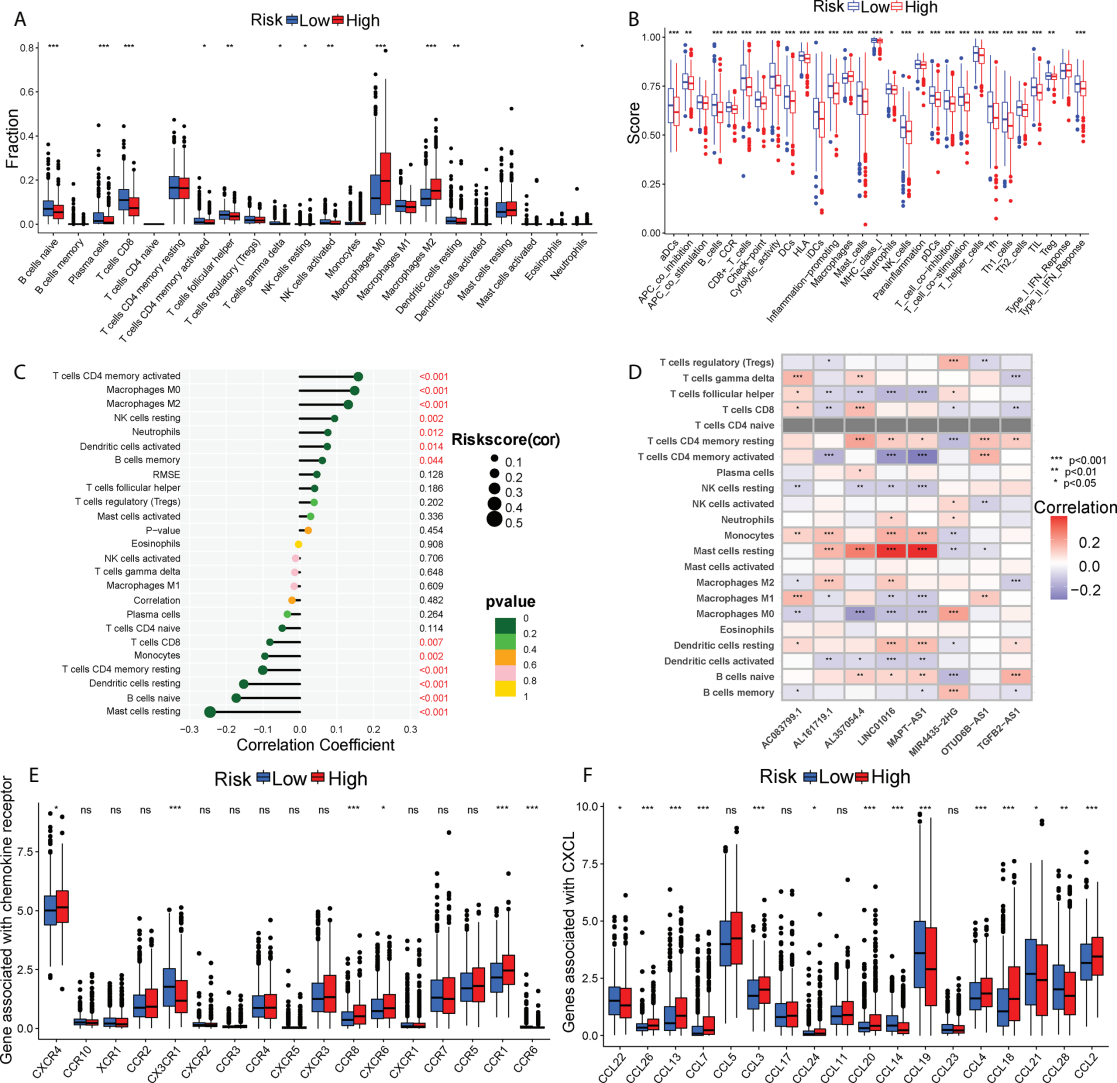
The immune landscape between two risk subgroups. **(A)** Differential immune infiltrates of 22 immune cell types. **(B)** 13 related immune pathways. **(C)** The correlation of risk score and immune cells. **(D)** The heatmap showing the correlation of 8 GT-lncRNAs with immune cells. The distinct expressions of chemokine receptors **(E)** and chemokines **(F)** between two risk groups. *P < 0.05, **P < 0.01; ***P < 0.001; ns, no significance.

**Figure 5:**
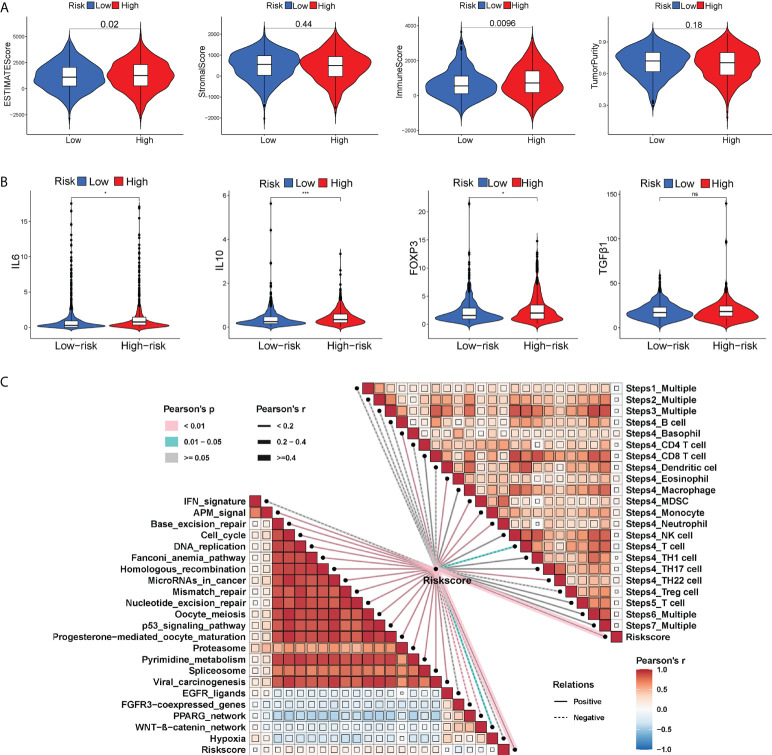
The differential immune activation between two risk subgroups. Violin plots of the ESTIMATE score, stromal score, immune score and tumor purity between two risk groups **(A)**. Violin plots showing the expression of immunosuppressive factors in the TME (IL6, IL10, FOXP3 and TGF-β) between two risk groups **(B)**. **(C)** Correlation of the risk score with cancer-immunity cycle and some biological progress in BC progression. *P < 0.05; ***P < 0.001; ns, no significance.

Tumor progression invariably involves the immune system. In fact, the type, location, density and functional orientation of different immune cell populations could profoundly affect the prognosis of different cancers (26). The distribution of 22 different immune cell types in high- and low-risk groups was examined using the CIBERSORT algorithm (**Figure 4A**). It was discovered that the immune cell infiltration was varied in the high- and low-risk groups. In comparison to the high-risk group, the low-risk group had significantly higher ratios of naive B cells, plasma cells, CD8+ T cells, memory activated CD4+ T cells, follicular helper T cells, gamma delta T cells, resting NK cells, activated NK cells, and resting dendritic cells, whereas the high-risk group had a higher infiltration of M0, M2 macrophages. Besides, the enrichment scores of immune-related functions generated by ssGSEA were also generally higher in BC patients with low-risk scores (**Figure 4B**). The risk score correlated positively with M0 and M2 macrophages and negatively with CD8+ T cells (P< 0.01), as shown in **Figure 4C**. The heatmap also displayed the association between 8 GT-lncRNAs and 22 immune cells (**Figure 4D**). Moreover, the variable immune infiltration levels, which may result in differing disease progression and immunotherapeutic efficacy, were further revealed by the unique differences in 14 out of 18 chemokines (77.77%) and 6 out of 17 chemokine receptor genes (35.29%) among two risk subtypes (**Figures 4E, F**). Immunological characterization appears to have been a significant factor in the risk score. Our analysis of the ESTIMATE algorithm confirmed that BC patients with high-risk scores also had higher immune scores and ESTIMATE scores (stromal score combined with immune score) (P< 0.05) (**Figure 5A**). The stromal score and tumor purity, however, did not show a discernible difference between the two risk groups. In addition, we compare the expression levels of TME immunosuppressive variables, such as IL-10, TGF-β, Treg marker FOXP3, and cancer-associated adipocytes activated marker IL-6 (27), across the two risk groups. Aside from TGF-β expression, high-risk patients had greater levels of IL-6, IL-10, and FOXP3 expression, indicating an intense immunosuppressive microenvironment, which may facilitate immune escape and a poor prognosis for BC patients in the high-risk group (**Figure 5B**). Immunity-mediated anti-tumor responses represent promising strategies for improving cancer patients’ long-term survival (28). A seven-step process known as the cancer-immunity cycle iterates and repeatedly expands to eradicate cancer cells (19). The risk score revealed a positive correlation with most steps in the cancer immunity cycle and most processes in cancer malignant progression (**Figure 5C**).

### Differential response profiles to ICI treatment

**Figure 6:**
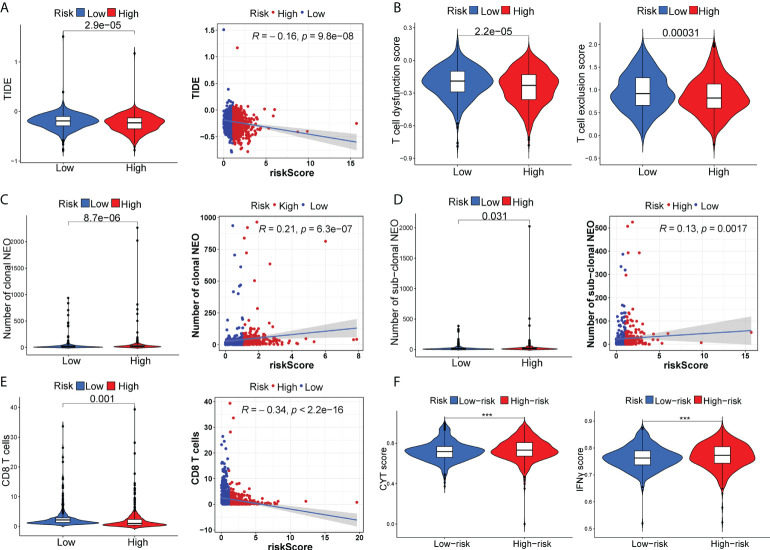
The distinct response to ICI treatment. The estimated TIDE score **(A)**, T-cell dysfunction score and T-cell exclusion score **(B)**, neoantigen burden **(C, D)**, CD8+ T cells **(E)**, CYT score and IFN-γ score **(F)** between two risk subgroups. ***P < 0.001.

**Figure 7:**
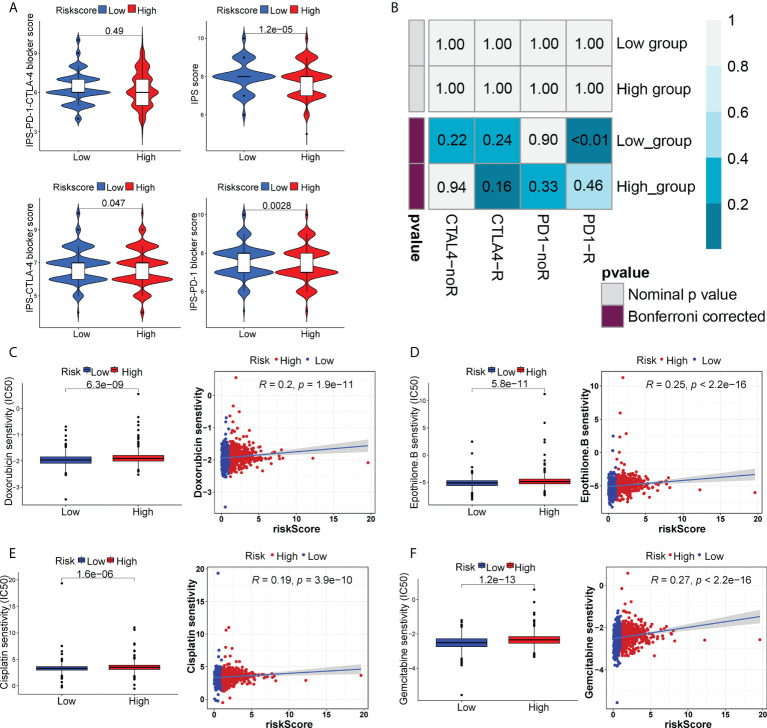
The distinct sensitivity to ICI therapy and chemo-drug therapy. **(A)** Four subtypes of IPS values (IPS score, IPS-PD1 blocker score, IPS-CTLA4 blocker and IPS-PD1-CTLA4 blocker). **(B)** The possible response to anti-PD1 and anti-CTLA4 immunotherapy in the two risk groups. Drug sensitivity of doxorubicin **(C)**, epothilone B **(D)**, cisplatin **(E)** and gemcitabine **(F)** in two risk groups.

The appliance of immune checkpoint inhibitors (ICIs), particularly anti-PD1/PD-L1 and anti-CTLA4, has revolutionized the treatment of cancer (29). CTLA4 expression was elevated in patients in the high-risk group, while PD-1 and PD-L1 expression appeared to be indistinguishable between the two risk groups (**Figures S6A–C**). TIDE, a method to uncover factors that underlie mechanisms of tumor immune escape, could operate as an effective biomarker in predicting immunotherapy response in patients with diverse malignancies, notably those treated with ICIs (30). A higher tumor TIDE prediction score is associated with worse ICB response as well as worse patient survival receiving anti-PD1 and anti-CTLA4 therapies (30). Our study found a substantial negative correlation between the risk score and the TIDE score (P< 0.001, | r | > 1), and BC patients in the high-risk group significantly got a higher TIDE score (P< 0.001) (**Figure 6A**). Especially, the low-risk group patients got higher both T-cell dysfunction score and T-cell exclusion score (P< 0.01, **Figure 6B**). Moreover, BC patients in the low-risk group had elevated expression profiles of TAMs and CAFs (P< 0.05, **Figures S6G, H**), though the MDSC displayed no significant differential expression patterns (P > 0.05, **Figure S6F**). These results revealed that BC patients had high-risk scores might evasde immune system through dual T cell dysfunction and exclusion strategies. Tumor neoantigens are frequently the target of effective adaptive immune responses against cancer cells, and in numerous malignancies, a higher neoantigen burden has been associated with better checkpoint blockade therapeutic outcomes (31, 32). Intriguingly, a significantly higher number of clonal and subclonal neoantigens was found in high-risk individuals (P< 0.01) (**Figures 6C, D**). Moreover, the degree of CD8+ T cell infiltration is tightly correlated with anti-tumor effects (33). In our study, the risk score was negatively correlated with the infiltration level of CD8+ T cells (| r | > 0.3, P< 0.001) (**Figure 6E**). Meanwhile, the Kaplan-Meier curves unraveled that a lower infiltration of CD8+ T cells predicted a worse OS (**Figure S6D**) and BC patients with high-risk scores and low infiltration level of CD8+ T cells had the shortest survival time (**Figure S6E**), demonstrating that the infiltration of CD8+ T cells may serve as a protective factor in BC prognosis. In the TME, activated T cells can release IFN-γ, a crucial cytokine that coordinates the innate and adaptive immune response against tumors (34). A direct effect of IFN-γ signaling on tumor and stromal cells is to upregulate the ligands PD-L1 and PD-L2, which bind with PD-1 on tumor-infiltrating T cells, thus suppressing the cytotoxic response. By using the ssGSEA method, we found that BC patients with high-risk scores had higher IFN-γ scores than those with low-risk scores (**Figure 6F**). Additionally, in certain tumor types, genes associated with immune cytolytic activity (CYT) could serve as a predictor of the clinical response to checkpoint blockade (35). Our results discovered a higher CYT score in high-risk patients (**Figure 6F**). Notably, the immunogenicity of tumor cells is a critical factor in determining the efficacy of ICIs, with tumors with increased immunogenicity being more responsive to ICIs (36). The relatively elevated levels of IPS-PD-11/CTLA4-blocker score in the high-risk patients indicated that individuals with high-risk scores might have higher tumor immunogenicity (**Figure 7A**). However, the Submap analysis uncovered that patients in the low-risk group responded more positively to anti-PD-1 treatment, whereas there was no response difference in anti-CTLA4 therapy (**Figure 7B**). In light of these findings, the risk score was a promising tool for stratifying BC patients with varying immunotherapy responses, and BC patients with high-risk scores might benefit more from the ICI treatment.

### Chemotherapy response prediction, TMB and mutational landscape

We also analyzed the sensitivity to certain chemo-drugs between two risk groups. The IC50 value of doxorubicin, epothilone B, cisplatin and gemcitabine was generally lower in the low-risk group, suggesting a better sensitivity to these drugs in low-risk patients (**Figures 7C–F**).

**Figure 8:**
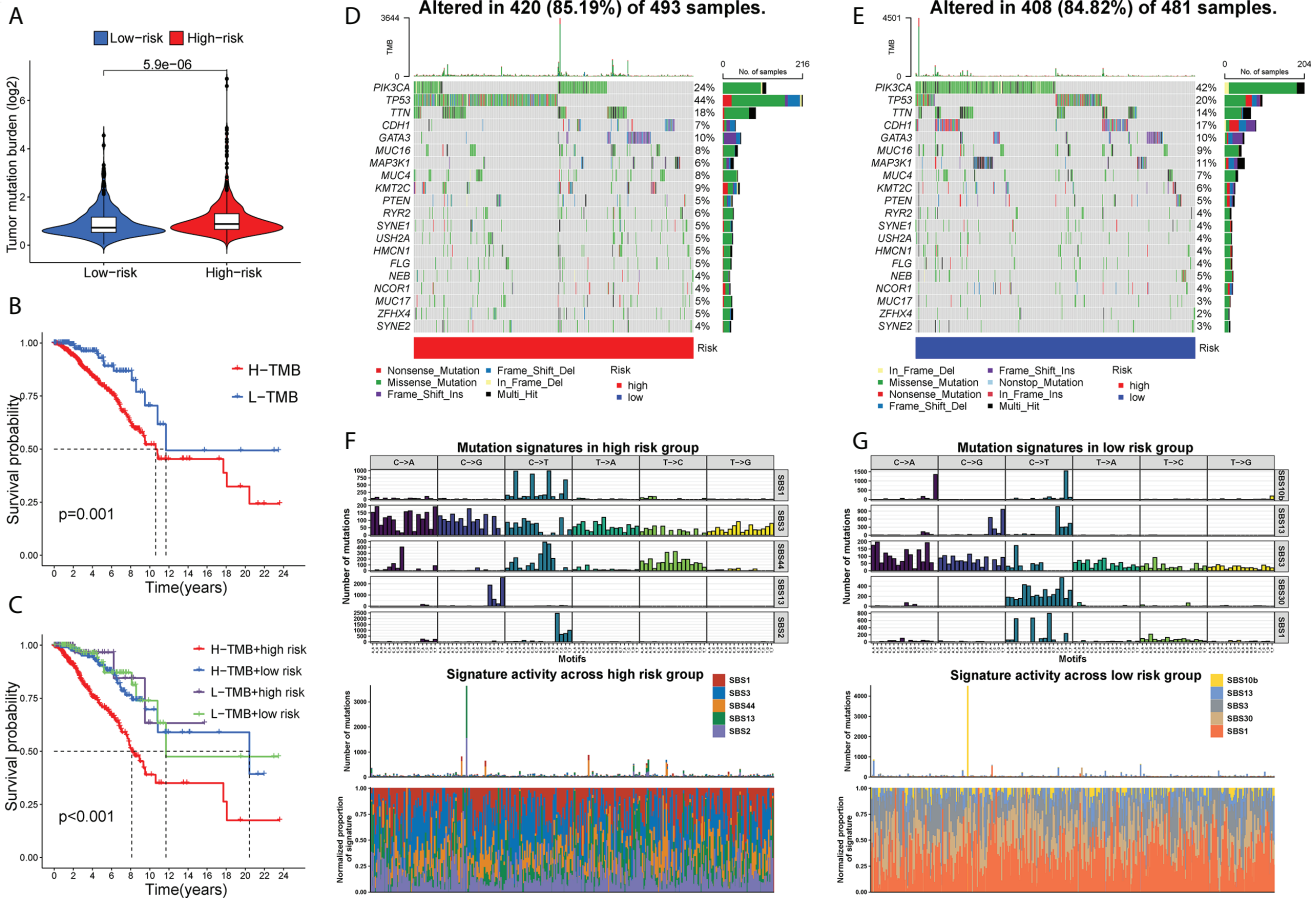
TMB and mutational profiles. **(A)** The estimated TMB score between two risk groups. The Kaplan-Meier curves showing the OS of BC patients under different TMB scores **(B)** and under different combinations of the risk score with TMB scores **(C)**. The mutational landscape of high- **(D)** and low-risk **(E)** patients. **(F, G)** The mutation signatures in two risk groups.

TMB, a reflection of total neoantigen load, has been identified as an effective biomarker for predicting ICI response (37). Consistently, BC patients in the high-risk group might benefit more from ICI therapy, unraveled by elevated TMB than those in the low-risk group (P< 0.001, **Figure 8A**). Moreover, a higher TMB combined with a higher risk score predicted a worse OS (**Figures 8B, C**). Then, we further analyzed the gene mutational distributions between two risk subgroups. TP53 had the highest genetic alteration (44%) in high-risk patients, whereas the highest genetic alteration gene in low-risk patients was PIK3A (44%) (**Figures 8D, E**), which may contribute to the distinct immunotherapy response patterns. Additionally, we identified mutational signatures in the COSMIC database by extracting data from genotype-specific somatic mutations of GT-lncRNAs. The results suggested that SBS3 was an independent feature in both high- and low-risk groups (**Figures 8F, G**), suggesting a possible association of the mutation profiles with defects in DNA-DSB repair by HR. In addition, the high-risk group was characterized by SBS44 while the low-risk group was characterized by SBS1 (**Figures 8F, G**). These findings showed that the mutation pattern of low-risk patients might correlate with spontaneous or enzymatic deamination of 5-methylcytosine.

### MIR4435-2HG affected BC cell proliferation and migration, macrophage polarization and PD-1/PD-L1/CTLA4 expression

**Figure 9:**
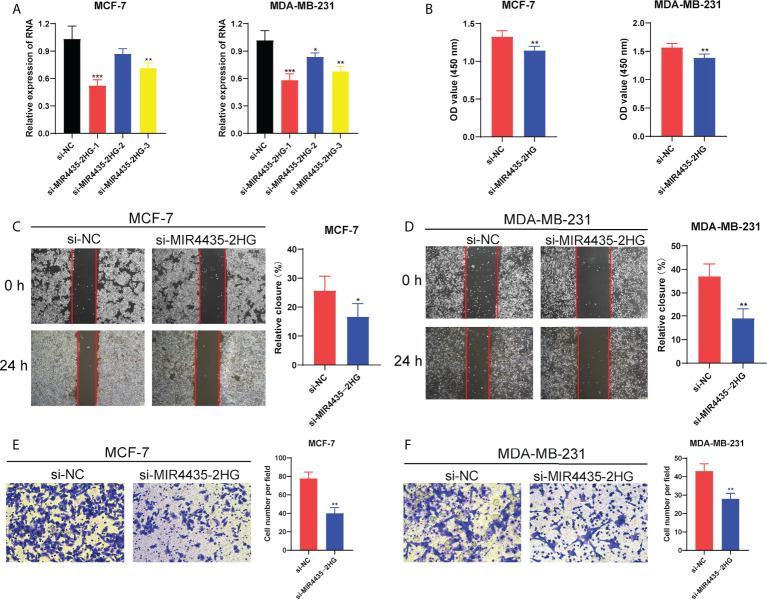
MIR4435-2HG affected BC cell proliferation and migration. **(A)** The RNA expression level of MIR4435-2HG in MCF-7 and MDA-MB-231 cells after silencing. **(B)** The CCK-8 assay showing the proliferation ability of MCF-7 and MDA-MB-231 cells after MIR4435-2HG knockdown. The scratch/wound healing and transwell assay showing the migration of MCF-7 cells **(C, E)** and MDA-MB-231 cells **(D, F)** after silencing MIR4435-2HG. *P < 0.05; **P < 0.01; ***P < 0.001.

**Figure 10:**
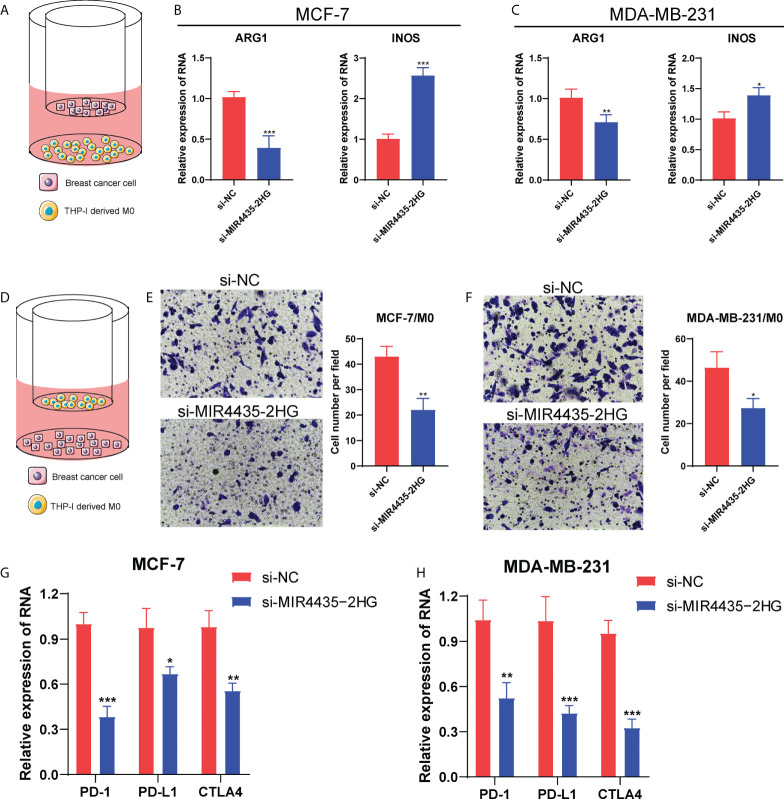
MIR4435-2HG affected macrophage polarization and PD-1/PD-L1/CTLA4 expression. The diagram for the co-culture between BC cells and THP-1-derived M0 macrophages **(A, D)**. The mRNA levels of ARG1 and iNOS in MCF-7 **(B)** and MDA-MB-231 cells **(C)**. **(E, F)** The transwell assay showing the migrated ability of macrophages after co-cultured with si-MIR4435-2HG BC cells. **(G, H)** The mRNA level of PD-1/PD-L1/CTLA4 in BC cells after MIR4435-2HG knockdown. *P < 0.05; **P < 0.01; ***P < 0.001.

Given that BC patients with high expression of MIR4435-2HG had a shorter survival time, we further explore the function of MIR4435-2HG in BC progression. Firstly, the RT-PCR analysis showed that the expression of MIR4435-2HG was significantly decreased in MCF-7 and MDA-MB-231 cells after transfection with siRNAs (**Figure 9A**). Then, the CCK-8 assay confirmed that compared with si-NC, the proliferation of MCF-7 and MDA-MB-231 cells under 24h were significantly suppressed *via* restraint of MIR4435-2HG (**Figure 9B**). Meanwhile, the scratch/wound-healing and transwell assays presented significant inhibition of migration abilities of MCF-7 and MDA-MB-231 cells by MIR4435-2HG knockdown (**Figures 9C–F**). Then, human BC cell lines (MDA-MB-231, and MCF-7) after transfection with MIR4435-2HG were co-cultured with THP-1 cells for 48h (**Figures 10A, D**). The results found that in THP-1 derived macrophages, the siRNA-mediated MIR4435-2HG knockdown in BC cells down-regulated the relative mRNA levels of macrophage M2 marker (ARG1) and up-regulated the levels of macrophage M1 marker (iNOS) (**Figures 10B, C**). The transwell assay discovered that the migrated ability of macrophages was significantly inhibited by the knockdown of MIR4435-2HG in BC cells (**Figures 10E, F**). These results proposed that MIR4435-2HG might promote the migration of macrophages and facilitate the polarization of M1 into M2 macrophages, thus favoring BC progression. Moreover, the suppression of MIR4435-2HG also inhibited the mRNA levels of PD-1, PD-L1 and CTLA-4 in BC cells (**Figures 10G, H**).

## Discussion

BC is of high complexity featured with the diverse presentation, morphological, biological, and clinical phenotypes. Effective prognostic and predictive classification systems will be conducive to revealing the biological and clinical heterogeneity of BC (38). Glycoproteins are ideal serum biomarkers for their active secretion or leakage into the circulation from tissues or blood cells. Abnormal glycosylation in tumors derives from changes in glycosyltransferase gene expression (39). Moreover, the activities of glycosyltransferases differ markedly between normal and tumor cells and the expression of GTGs can be utilized to classify BC subtypes (40). This study used 8 GT-lncRNAs to identify two-risk subtypes, which exhibited different survival states, immune infiltration and immunotherapy response. BC cases were divided into high-risk and low-risk groups based on the median risk score, calculated by 8 GT-lncRNAs, including MIR4435-2HG, MAPT-AS1, TGFB2-AS1, AL357054.4, AL161719.1, OTUD6B-AS1, AC083799.1, and LINC01016. The risk score was significantly associated with OS, DFS and PFI of BC patients, whereby low-risk-group patients showed a better prognosis regardless of clinical characterization. Moreover, the risk score could serve as an independent predictor of BC prognosis, and the predictive ability and performance of the risk score gradually stabilized and surpassed age, clinical stage and TNM stages over time increased. Generally speaking, the risk score can evaluate high-risk BC patients and identify individual survival probability.

MIR4435-2HG is an oncogene that participates in EMT, tumor cell proliferation, apoptosis, migration, and invasion (41). Downregulation of MR4435-2HG was reported to inhibit BC progression *via* the Wnt/β-catenin signaling pathway (42). Our *in vitro* validation showed that the MIR4435-2HG knockdown significantly inhibited the proliferation and migration of BC cells, which is consistent with previous reports. OTUD6B-AS1 is a newly identified lncRNA, whose overexpression could suppress tumor growth in kidney cancer, thyroid cancer, and colorectal cancer, but boost tumor growth in hepatocellular carcinoma (43). Ma et al. reported OTUD6B-AS1 as an immune-related lncRNA and served as a risk factor in BC patient prognosis (44). Likewise, OTUD6B-AS1 was classified as a ferroptosis-related risk lncRNA in BC survival (45). MAPT-AS1 is at the anti-sense strand of the MAPT promoter region to regulate MAPT, and the overexpression of MAPT-AS1 was verified to predict better BC patient survival (46). TGFB2-AS1 is associated with tumorigenesis, and promotes migration and invasion of HepG2 cells (47), but inhibits migration and invasion of lung adenocarcinoma cells (48). Regrettably, the biological functions and prognostic presentation of TGFB2-AS1 in BC are still unclear. AC083799.1 is only reported as an autophagy-related lncRNA and a protective factor for endometrial cancer patient prognosis (49). LINC01016 is uncovered as a direct transcriptional target of ERα and displayed positive clinical outcomes for BC patient prognosis (50). There are yet no reports about AL161719.1 and AL357054.4. Collectively, 4 of our 8 lncRNAs were associated with BC survival state and our study firstly identified the prognostic value of other 4 GT-lncRNAs, namely AL161719.1, TGFB2-AS1, AL357054.4 and AC083799.1 in BC patient survival.

The construction of predictive models based on GTGs has been reported for cancer prognosis. For instance, a glyco-signature combined 19 glycosyltransferases could stratify pancreatic ductal adenocarcinoma patients with different clinic prognostic outcomes (51). Pan et al. performed a glycoproteomic study, finding that the intact glycopeptide signature could serve as a survival predictor for patients with high-grade serous ovarian carcinoma (52). In general, these reports highlight the underlying efficiency of glycosylation-based signature in predicting cancer prognosis. In our previous study, we successfully established a 9-gene signature under glycosylation characteristics for predicting BC prognosis, and BC patients with different outcomes obviously got different risk scores (15). Based on this, we further constructed an 8-GT-lncRNA signature. Compared with prognostic signatures of ours and others in BC, the present signature under GT-lncRNA patterns displayed a better performance and predictive ability as the AUC value of 3-year OS was the highest. Hence, the combination of glycosylation and lncRNA expression profiles might provide novel insights into predicting the prognosis of patients with various tumors.

TME is closely associated with tumorigenesis and yields signals relevant to tumor prognosis and the prediction of immunotherapy response (53). Immune checkpoint inhibitors (ICIs) are monoclonal antibodies (mAbs) that stimulate the immune system by suppressing the delivery of co-inhibitory signals (54). The primary targets for ICIs mainly include PD-1, PD-L1 and CTLA4. PD-1 is a receptor mainly expressed on the surface of activated T cells. The cross-linkage of PD-1 to its ligand PD-L1 induces non-reactivity of T cells, which is the key mechanism of tumor immune tolerance for tumor cells PD-L1 overexpression (54). CTLA4 is a negative immune regulator, which restricts T cell activation *via* various suppressive functions such as competition with CD28, regulation of Treg cells, and the control of adhesion and motility (55). High intra-tumoral PD-1+ and CD8+ cell density were reported to be associated with the improved recurrence-free survival of rectal cancer patients, and high intra-tumoral CD8+ cell density predicted a better OS of rectal cancer patients (56). Likewise, Fang et al. reported that a higher frequency of TCF-1+PD-1+CD8+ T cells was significantly related to the beneficial response to PD-1 blockade (57). Therefore, the infiltration of CD8+ T cells and expression of ICI-related genes was a good indicator in assessing response to ICI treatment. The present study observed that low-risk-groups patients were enriched with most immune cell infiltrations, got higher scores for each step in the cancer-immunity cycle, and especially had higher infiltration of CD8+ T cells. Moreover, PD-1/PD-L1 and CTLA4 were significantly highly expressed in the low-risk group both in the bioinformatics and clinical samples. The Submap analysis showed that patients in the low-risk group responded more positively to anti-PD-1 treatment, whereas there showed no responsive difference in anti-CTLA4 therapy. Our data demonstrated that the risk score may act as a predictive marker for response to ICI therapy in BC.

Nevertheless, this study still has some limitations. Firstly, this study is a retrospective study based on bioinformatics analysis from the online database, lacking prospective external validations. Secondly, we just only validated the biological function of MIR4435-2HG in BC proliferation and migration. The underlying mechanism of those GT-lncRNAs in BC tumorigenesis, progression, and prognosis still need further exploration. Lastly, it is more meaningful and reliable to validate the predictive ability of the risk score in a large and multicenter cohort.

## Conclusion

In conclusion, the present study identified and verified a robust 8-lncRNA signature that was an independent prognostic factor for BC patient prognosis and exhibited superior predictive performance to previous models. Notably, our signature identified different immune infiltration, immunotherapy responsiveness, and chemo-drug sensitivity between two risk subtypes. This will be a promising supplementary predictive means for promoting BC treatment.

## Data availability statement

The original contributions presented in the study are included in the article/**Supplementary Material**. Further inquiries can be directed to the corresponding authors.

## Author contributions

QZ, JZ, and YW conceived and supervised the project. WL conducted experiments and analyzed the data. YT wrote and revised the manuscript. XZ conducted the literature investigation. All authors contributed to the article and approved the submitted version.

## Funding

This work was supported by China Guanghua Science and Technology Foundation (grant number: 2019JZXM001) and Wuhan Science and Technology Bureau (grant number: 2020020601012241).

## Conflict of interest

The authors declare that the research was conducted in the absence of any commercial or financial relationships that could be construed as a potential conflict of interest.

## Publisher’s note

All claims expressed in this article are solely those of the authors and do not necessarily represent those of their affiliated organizations, or those of the publisher, the editors and the reviewers. Any product that may be evaluated in this article, or claim that may be made by its manufacturer, is not guaranteed or endorsed by the publisher.

## Abbreviations

BC, breast cancer; CCK-8, Cell Counting Kit-8; C-index, concordance index; CTLA4, cytotoxic T-lymphocyte antigen4; DFI, disease-free interval; DMEM, Dulbecco’smodified Eagle’smedium; EMT, epithelial-mesenchymal transition; FDR, false discovery rate; GO, Gene ontology; GSEA, gene set enrichment analysis; GTGs, glycosyltransferase-related genes; GT-lncRNAs, glycosyltransferase-related lncRNAs; IC50, half-inhibitory concentration; ICIs, immune checkpoint inhibitors; IHC, immunohistochemistry; IPS, immunophenoscore; KEGG, Kyoto Encyclopedia of Genes and Genomes; lncRNAs, long non-coding RNAs; NMF, nonnegative matrix factorization; OD, optional density; OS, overall survival; PBS, phosphate-buffered saline; PD-L1, programmed cell death ligand-1; PD-1, programmed cell death-1; PFS, progression-free survival; ROC, receiver operating characteristic; RMS, restricted mean survival; siRNAs, small interference RNAs; TCIA, The Cancer Immunome Atlas; TOM, topological overlap measure; TME, tumor microenvironment; WGCNA, weighed gene co-expression network analysis.
