# Supplementary material for: Landscape of prognosis and immunotherapy responsiveness under tumor glycosylation-related lncRNA patterns in breast cancer
Source: Front Immunol. 2022 Sep 15;13:989928. doi: 10.3389/fimmu.2022.989928 (PMC9520571; doi:10.3389/fimmu.2022.989928)

**Supplementary materials**

# Supplementary Figures

## Figure S1. Construction of the risk score. (A) Lasso coefficient profiles of prognostic GT-lncRNAs. (B) Multivariate Cox regression analysis selected 8 GT-lncRNAs to construct a risk signature.


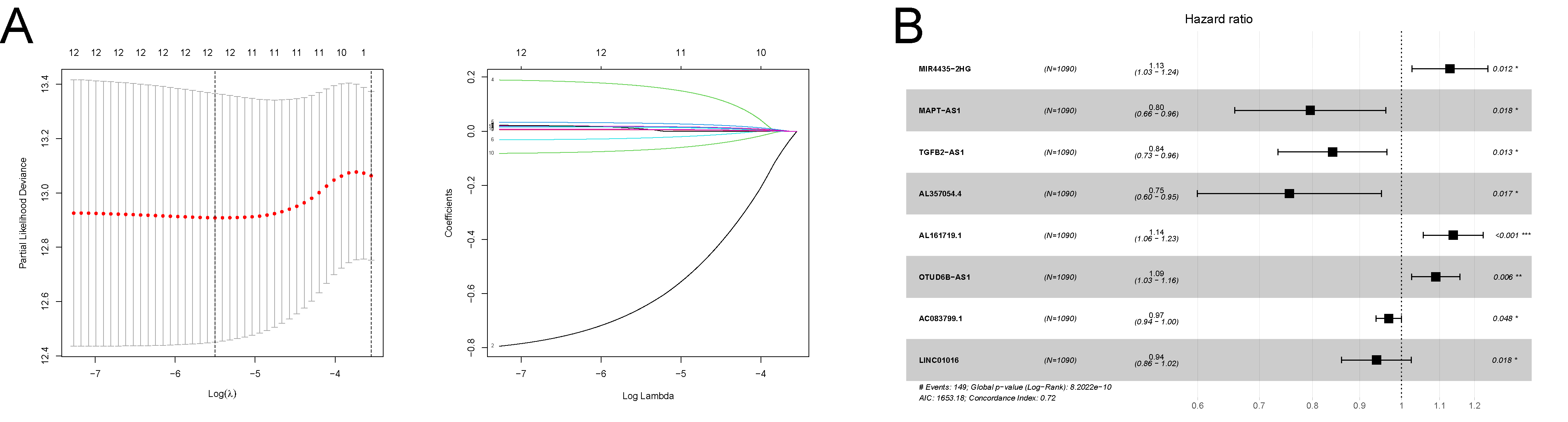


## Figure S2. Prognostic value of the risk score. The Kaplan-Meier curves of high- and low-risk-group patients with different OS, DFS and PFI in the training set (A) and testing set (B).


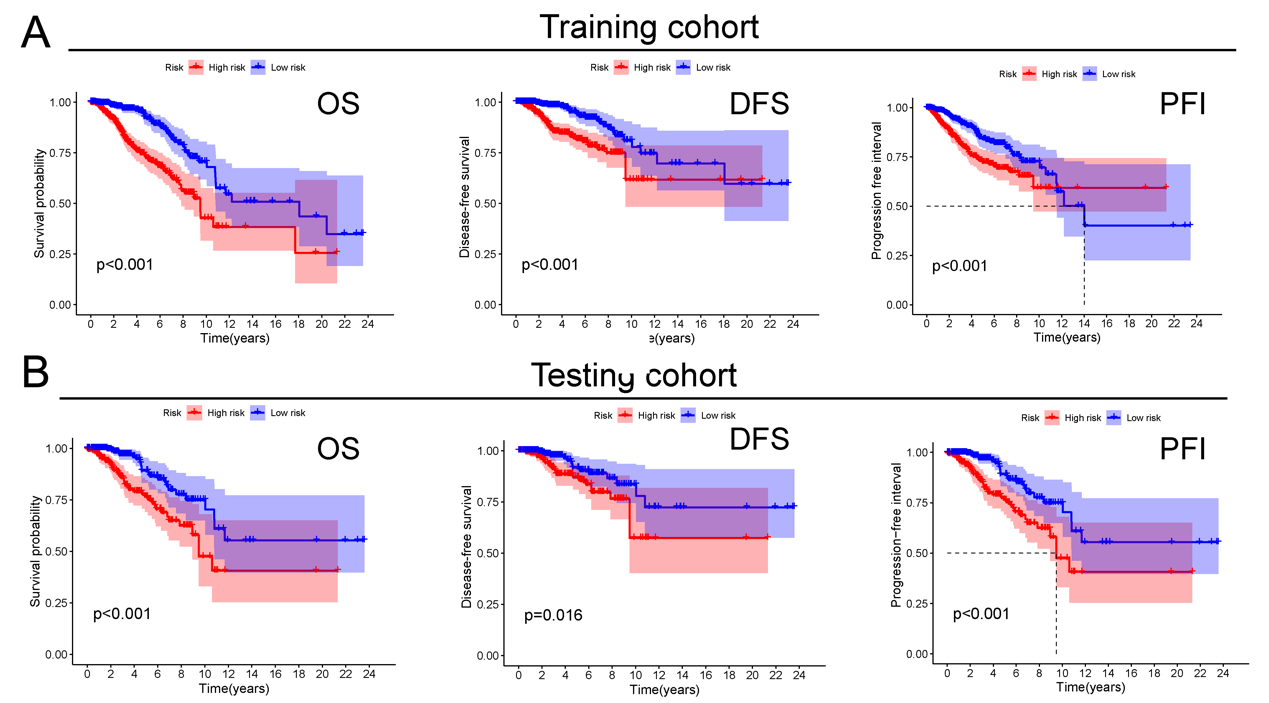


## Figure S3. Validation of the risk score in the testing set. Nomogram model based on the risk score and other clinical factors and calibration plots of the nomogram for internal validation in the training set (A) and testing set (B). (C) ROC curves for the risk score at 1-, 2- and 3- year. (D) ﻿ROC curves for the risk score, age, gender, clinical stage, and TNM stages at 1-year. (E) The time-dependent C-index curve of the risk score, age, gender, clinical stage, and TNM stages.


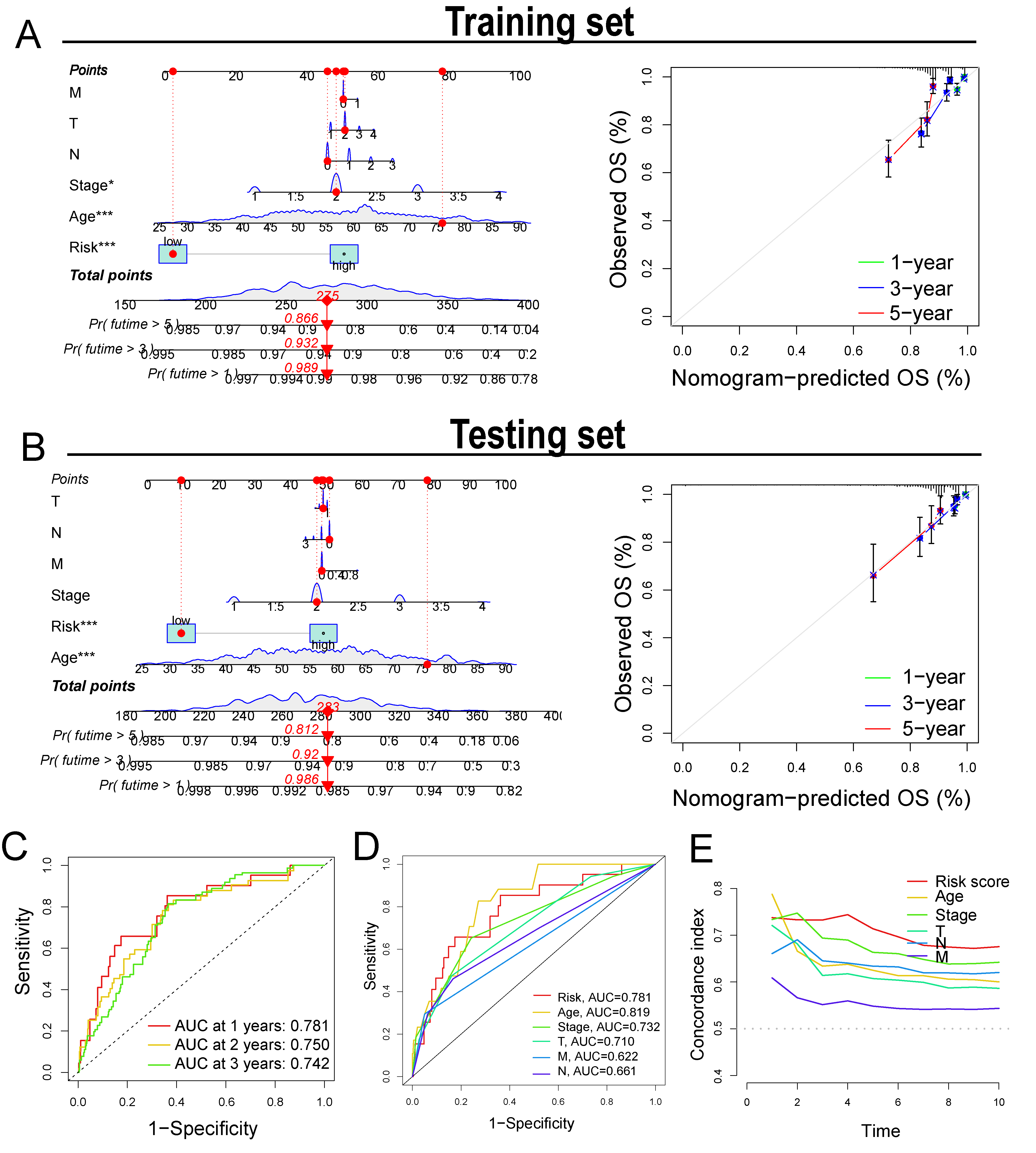


## Figure S4. The external comparison of models. ﻿The ROC curve of Ping’s (A), Luo’s (B), Zhang’s (C), and Zhao’s (D) signatures. The Kaplan-Meier curves of two-risk-group patients’ OS in Ping’s (E), Luo’s (F), Zhang’s (G) and Zhao’s (H) signatures. (I) ﻿C-index comparison of 5 prognostic risk models. (J) ﻿Restricted mean survival (RMS) curves for 5 risk models.


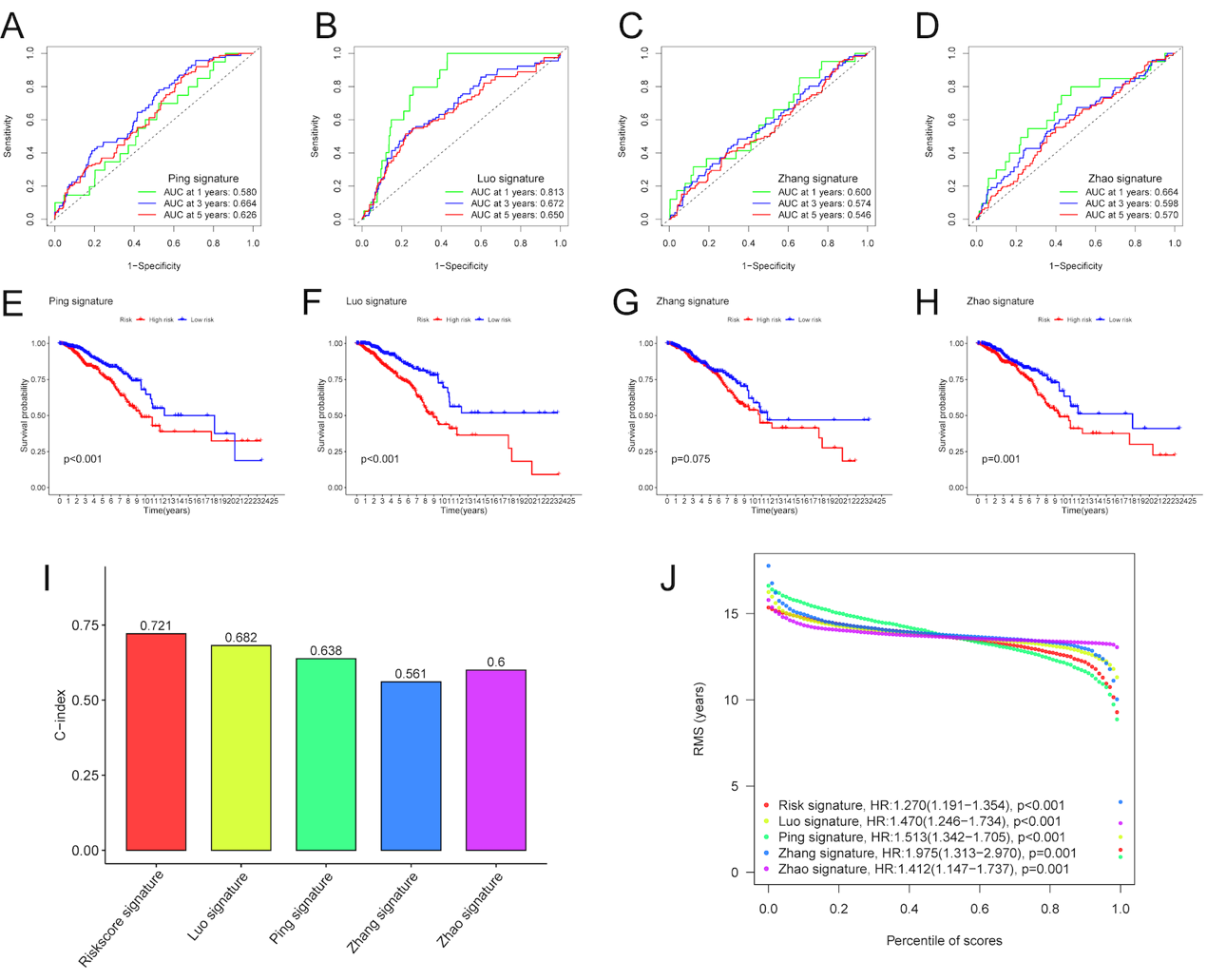


## Figure S5. The OS of BC patients with different subtypes in the high- and low-risk group.


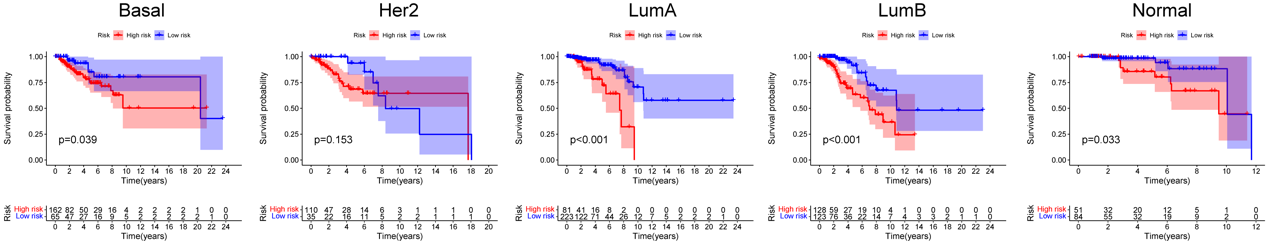


## Figure S6. The immunotherapy landscape between two risk subgroups. The estimated PD-1 (A), PD-L1 (B) and CTLA4 (C) between two risk subgroups. The Kaplan-Meier curves showing the OS of BC patients with high- and low- CD8+ T cells (D) and with different ﻿combinations of risk scores and CD8+ T cell infiltrations (E).


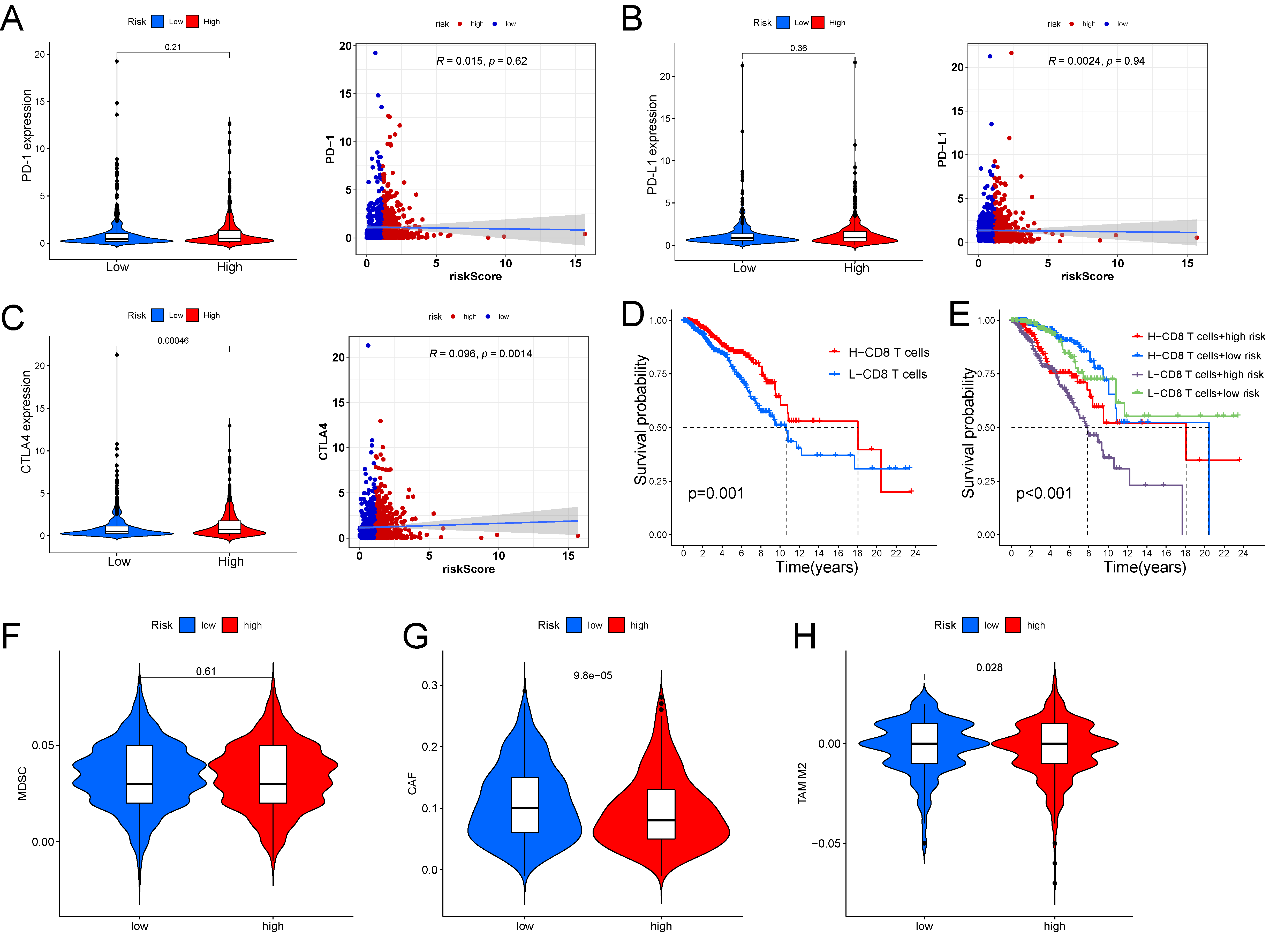

Supplement: Supplementary file 1 [file DataSheet_1.docx]
